# Exploring the role of spiritual leadership among nurse colleagues: an associative analysis of its impact on passion and altruism

**DOI:** 10.1186/s12912-025-02750-5

**Published:** 2025-02-07

**Authors:** Huda Gaber Hamzaa, Mohamed Hussein Ramadan Atta, Heba Mostafa Ali Taha, Mervat Amin Sayed, Asmaa Kamal Ahmed, Ahmed Abdellah Othman, Nadia Mohamed Ibrahim Wahba

**Affiliations:** 1 Faculty of Nursing, Psychiatric Nursing and Mental Health, Port Said University, Port Said City, Egypt; 2 Nursing Department, College of Applied Medical Sciences, Prince Sattam Bin Abdulaziz University, Wadi Addawasir, Saudi Arabia; 3 Psychiatric and Mental Health Nursing Department, Faculty of Nursing, Alexandria University, Alexandria, Egypt; 4 Nursing Administration, Faculty of Nursing, Assiut University, Assiut City, Egypt; 5 Community Health Nursing, Fayoum University, Fayoum City, Egypt; 6 Nursing Administration, Faculty of Nursing, Fayoum University, Fayoum City, Egypt; 7 Nursing Administration, Faculty of Nursing, Sohag University, Sohag City, Egypt; 8 College of Nursing, Prince Sattam Bin Abdulaziz University, Al-Kharj City, Saudi Arabia

**Keywords:** Altruism, Leadership, Nurses, Passion, Spirituality

## Abstract

**Background:**

Spiritual leadership emphasizes that nurses encounter situations that require compassion, empathy, and a deep understanding of the human experience. Passion can drive nurses' motivation, engagement, and commitment to patient care. Altruism is a core value in nursing that involves selfless concern for the well-being of others. Spiritual leadership might be an essential organizational resource in enhancing followers’ inspiring vision, compassion for others, and passion for their work.

**Aim of the study:**

To investigate the role of spiritual leadership in the relationship between altruism and passion for work among nurse colleagues.

**Subjects & method:**

A multicenter descriptive, cross-sectional research study was conducted at six Nursing Egyptian faculties affiliated with Alexandria, Port-Said, Fayoum, Damanhur, Assiut, and Sohag Universities, with 391 nurse colleagues.

**Data collection tools:**

Personal and Job-related Data Questionnaire, Spiritual Leadership Questionnaire (SLQ), Passion Scale, and 9- Self-Report Altruism Scale (9- SRA) were utilized to collect data.

**Results:**

The current study verified that nurse colleagues had high levels of perceived spiritual leadership, passion, and altruism (50.9, 47.6, and 40.2, respectively). The results revealed noteworthy positive correlations between spiritual leadership, passion, and altruism (*r* = 0.644, 0.519, & 0.509). Furthermore, mediation analysis highlighted that spiritual leadership could mediate the effect of altruism on the passion level.

**Conclusion:**

This study delivers valued insights into the crucial role of spiritual leadership in augmenting nurse colleagues’ altruistic tendencies and passion.

**Recommendations:**

Cultivating workplace spirituality on a daily agenda is a key component of management that requires spiritual competencies from nurse leaders. These competencies ultimately enhance passion for work, performance, and altruistic behaviors.

**Supplementary Information:**

The online version contains supplementary material available at 10.1186/s12912-025-02750-5.

## Introduction

Spirituality is an individual and subjective facet of human experience that involves the search for meaning, purpose, and a connection to something beyond oneself, whether through organized religion, personal beliefs, nature, or existential reflections [1, 2]. It serves as a source of inner peace and transcendence, encompassing diverse beliefs and practices [3]. However, challenges in spiritual leadership, passion, and altruism among nurses have been observed both within the research community and globally, raising concerns about their potential impact on the nursing profession. These issues may affect nurses' ability to maintain emotional balance, a positive outlook, and a sense of interconnectedness with others, ultimately influencing their sense of well-being and ethical decision-making [4]. Addressing such challenges is critical, as spirituality in nurses can enhance their overall quality of life, contribute to psychological and emotional well-being, and provide a framework for understanding and navigating the complexities of the human experience, personally and professionally [5].

Spiritual leadership in nursing involves a comprehensive approach beyond conventional managerial duties. It revolves around guiding and motivating individuals in the healthcare setting by incorporating spiritual values, ethical principles, and a sense of purpose into leadership practices. This leadership style recognizes the interdependence of physical, emotional, and spiritual aspects of well-being, underlining the importance of addressing the spiritual needs of both patients and healthcare providers [1]. Spiritual leadership to cultivate nurses’ workplace environment that nurtures empathy, passion, and a profound understanding of the human experience [2]. It acknowledges the inherent worth of each person and fosters a sense of community and interconnectedness among the healthcare team [3].

Nurse leaders may have a sense of purpose in their teams, positively impacting job satisfaction, resilience, and overall welfare. It was documented in several studies that nurses have a moderate level of spiritual leadership [5], ranging from mild to high (6). Furthermore, spiritual leadership improves patient care by acknowledging and honoring patients' spiritual beliefs and values, fostering a more patient-centered and culturally aware healthcare approach. Ultimately, spiritual leadership in nursing plays a crucial role in creating a holistic and empathetic healthcare environment [4].

Spiritual leadership, as a concept, comprises two primary components; the first component focuses on performance and includes three critical subscales: commitment, vision, and productivity [5]. Commitment refers to a leader's dedication to ethical and moral principles, guiding their decision-making and actions. Vision involves articulating and inspiring others with a shared purpose and direction. Productivity reflects the leader's commitment to achieving organizational goals efficiently and effectively [6]. The second component centers around attendance and involves two vital ingredients: belonging and belief. Belonging highlights creating a supportive and inclusive environment where team members feel connected and valued. Belief pertains to fostering a shared belief system that aligns with the organization's values, promoting unity among team members. This comprehensive framework, with its dual components and five specific subscales under each, forms the foundation for nursing spiritual leadership, providing a holistic approach to leadership that goes beyond traditional managerial aspects [7, 8].

Spiritual leadership emphasizes nurses possessing a deep sense of purpose, guided by ethical values and committed to the well-being of patients and the healthcare team. This sense of purpose aligns with a nurse's passion for making a positive impact on the lives of others [3]. Additionally, spiritual leadership emphasizes the importance of empathy and compassion, encouraging nurses to connect with patients holistically and acknowledging their physical, emotional, and spiritual needs. This empathetic approach resonates with a nurse's innate passion for providing patient-centered care beyond clinical procedures. Furthermore, spiritual leaders in nursing foster a collaborative and supportive work environment, recognizing the interconnectedness of the healthcare team. This cooperative spirit aligns with a nurse's passion for teamwork and a shared commitment to improving patient outcomes [9, 10].

Fry [11]. Incorporated spirituality as a long-neglected aspect into leadership theories and ultimately proposed the concept of spiritual leadership, which emphasizes intrinsically motivating oneself and others through the leader’s values, attitudes, and behaviors. Conceptually, spiritual leadership comprises three principal components, vision, hope/faith, and altruistic love, as the leader’s values, attitudes, and behaviors.

Passion in nursing encapsulates an intense and genuine commitment to the profession, reflecting a deep love and enthusiasm for the intricacies of patient care. This enthusiasm is evident in nurses' unwavering dedication to their roles beyond job requirements. A study by Cleveland et al. [12] documented that nurse trustees demonstrate a strong passion for community service. Although survey participation rates vary by state, the respondents contribute to every state and even extend their efforts to some international communities. Passionate nurses exhibit behaviors such as continuous learning, the proactive pursuit of excellence, and a genuine concern for the well-being of their patients [13]. This commitment is reflected in their tireless efforts to stay abreast of medical advancements, provide empathetic and personalized care, and advocate for their patients' rights. Passion for nurses is a driving force that fuels resilience and determination in the face of the challenges inherent in healthcare. Passionate nurses inspire their colleagues, contribute to a positive work culture, and ultimately enhance the overall quality of patient care [14]. In addition, passion is a cornerstone of nursing practice, shaping the profession's character and fortifying the commitment to delivering compassionate and holistic care to those in need. Passion fuels the resilience and determination needed to navigate healthcare's complex and demanding nature. It propels nurses to go above and beyond their duties, fostering a culture of excellence and being altruistic to others [15]. A study by Gkorezis et al. [16] found that nurses have considerable passion.

Furthermore, Vallerand et al. [17] presented his Dualistic Model of Passion, demonstrating its importance and effects. They explained that it is a strong tendency of the individual toward an activity of high value, which the individual loves, spends time and energy on, finds happiness and luxury, and achieves a balanced and purposeful life. According to this model, passion includes two dimensions: the harmonious academic passion that contributes to the continuous happy engagement in academic work and helps to prevent experiences that negatively affect the individual due to psychological conflict and psychological discomfort, and the obsessive passion that controls experiences which negatively affect the individual as a result of psychological conflict and distress.

Altruism within the nursing context entails a selfless and compassionate dedication to the welfare of others, surpassing mere professional obligations, with moderate to high levels among nurses [18]. It manifests through sincere empathy, kindness, and caregiving in nurses' interactions with patients, families, and peers. Altruistic behavior in nursing involves prioritizing others' needs, offering emotional support, and advocating for the patient's best interests [19]. This generous commitment becomes particularly evident during challenging situations, where nurses consistently strive to alleviate suffering and enhance the well-being of those under their care. The importance of altruism for nurses is profound, influencing positive patient outcomes and enriching healthcare experience. Altruistic gestures foster trust and a sense of security in the nurse-patient relationship and contribute to a positive work environment by strengthening teamwork and cultivating a compassionate culture. Ultimately, altruism is an inherent and essential aspect of nursing practice, shaping the profession's character and upholding the ethical principles guiding nurses in delivering comprehensive and patient-centric care [20, 21].

A higher altruism level was associated with a better quality of nursing. In addition, nurses responding to patients' ethical requirements can demonstrate professionalism and let the patient feel more thoroughly cared for [22]. Altruistic care is important in building a strong, trusting relationship that can reduce conflicts. Conversely, when altruistic care decreases, nursing quality also declines, with low nursing satisfaction and a high complaint rate [17].

Several studies have highlighted the importance of spiritual leadership in various contexts. Wang et al. [10] revealed the mediating role of calling in leadership with harmonious passion among managers. Additionally, Abou Zeid et al. [1] showed a strong positive correlation between spiritual leadership and psychological capital among nursing educators. Wu and Lee [3] found that spiritual leadership positively influences work engagement, with spiritual well-being and psychological capital mediating this effect. Other studies, such as that by Birnie [23], have shown that spiritual leadership can increase retention rates and improve the psychological well-being of nurses. These findings underscore the gap in knowledge regarding spiritual leadership among nurse colleagues and its potential association with their passion and altruism.

### Significance of the study and hypotheses development

The study examines the complex interplay between spiritual leadership, passion, and altruism in nursing, highlighting their collective influence on nurses' professional behavior, well-being, and patient outcomes. With its foundation in ethical values, vision, and a sense of purpose, spiritual leadership cultivates a workplace environment that nurtures resilience, compassion, and dedication among nurses [1, 3, 10]. This leadership style fosters a culture of inclusivity and interconnectedness, promoting the intrinsic motivation required for passionate and altruistic caregiving. Altruism, characterized by selfless concern for the well-being of others, and passion, defined as an intense commitment to patient care, are critical in enhancing nursing quality and reducing emotional exhaustion [12, 17, 19].

The study posits that spiritual leadership positively influences nurses’ passion and altruism, improving job satisfaction, psychological well-being, and patient-centered care. It also hypothesizes that passion mediates the relationship between spiritual leadership and altruistic behaviors, while spiritual leadership directly enhances the levels of passion and altruism among nurses [10, 11, 16]. By identifying these relationships, the study aims to provide evidence-based strategies for fostering compassionate and effective nursing practices and improving healthcare delivery and workplace dynamics. This research addresses a critical gap in understanding how spiritual and psychological frameworks can be leveraged to support the professional and emotional development of nursing staff [14, 23].

Hypotheses:

1. Spiritual leadership is positively associated with nurses' passion for work.
2. Spiritual leadership is positively associated with altruism among nurses.
3. Passion mediates the relationship between spiritual leadership and altruistic behavior in nursing.

### Setting and design

A multicenter descriptive, cross-sectional research study followed the "Improving the reporting of observational studies in epidemiology" (STROBE) checklist. The study was conducted at six Nursing Egyptian faculties affiliated with Alexandria, Port-Said, Fayoum, Damanhur, Assiut, and Sohag Universities. These universities have been selected to represent diverse Egyptian cultures; Fayoum University represents the center of Egypt, Damanhur University reflects the Delta and lower demographic region, Alexandria University represents the western region, Port-Said University is affiliated with the eastern and Suez Canal region, and Assiut and Sohag Universities affiliated to the upper Egypt region.

### Population and sample size calculation

The study focused on the entire population of nurse colleagues from all academic positions, comprising professors, assistant professors, lecturers, assistant lecturers, clinical demonstrators, and practical guides enrolled in the six chosen faculties. Data collection was conducted using a stratified random cluster sampling approach. Researchers utilized Open Epi, Version 3, an open-source calculator, to ascertain the necessary sample size. The total population size of nurse colleagues in the surveyed universities was 1504. Parameters for the calculation included a hypothesized percentage frequency of the outcome factor in the population (p) at 50%, with a ± 5% margin of error, a confidence level of 95%, a design effect (DEFF) of 1 (applicable for cluster surveys), and the formula for sample size calculation: Sample size (n) = [DEFF * N * p * (1-p)] / [(d^2 / Z^2) * (N-1) + p * (1-p)]. Based on these criteria, the minimum required sample size was 350. However, 30% of colleagues were included to accommodate potential non-response, the online data collection method, and the expected dropout rate. Consequently, the final sample size required for the study was 391 nurse colleagues.

### Sampling method

To recruit nurse colleagues, a dual-phase sampling approach was utilized. In the first phase, a stratified sampling method was employed to determine the number of nurse colleagues participating from each faculty. The intended sample sizes from the faculties of Alexandria, Port-Said, Fayoum, Damanhur, Assiut, and Sohag universities were 89, 54, 70, 58, 55, and 65, respectively.

#### Top of form

**Fig. 1:**
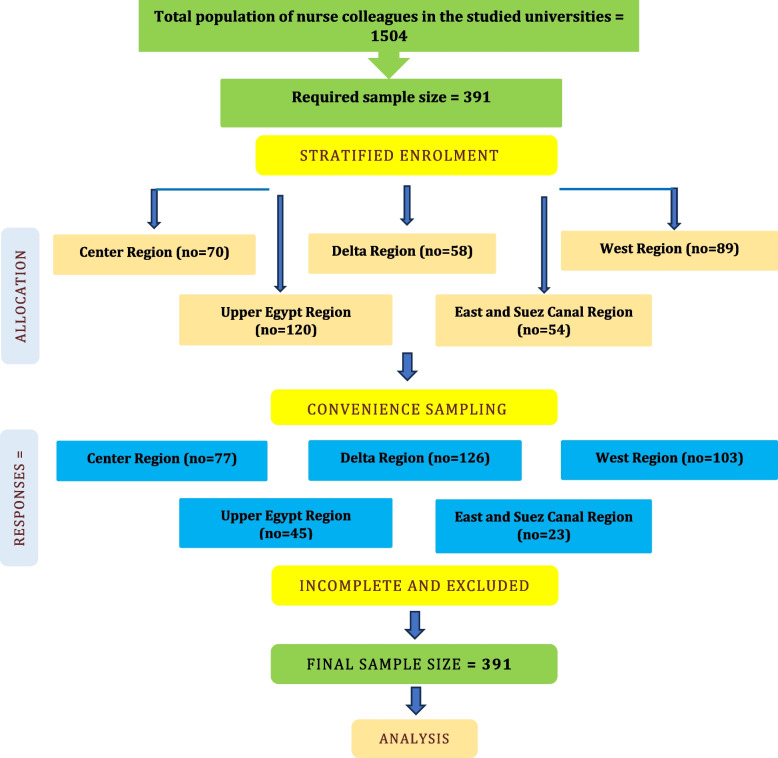
Flow sample

In phase 2, convenience sampling was used to recruit nurse colleagues in each faculty (Fig. 1). The inclusion criteria were as follows: nurse colleagues for at least six months ago enrolment, presently teaching or offering educational services for nursing students, full-time work engagement at a minimum of 20 h of work weekly, and revealing an interest and willingness to share in the study.

### Data collection instruments

The researchers utilized a four-section-based questionnaire to gather data:

#### Section I: nurse’ personal and job-related data questionnaire

The researchers established this structured questionnaire in Arabic. It comprised questions that elicited the studied nurse colleagues' personal and job-related characteristics, such as age, sex, marital status, residence, educational qualifications, years of nursing experience, and experience in a current position or working department (supplementary 1).

#### Section II: Spiritual Leadership Questionnaire (SLQ)

This questionnaire was established in English by Fry et al. [24]. I aim to assess spiritual leadership levels among nursing colleagues. SLQ was translated into Arabic by Abou Zeid et al. [1]. The SLQ contained 17 items classified into three dimensions: vision, hope/faith (five items for each), and generous love (seven items). The example of items encompassed, "I understand and am committed to my organization’s vision." and "I set challenging goals for my work because I have faith in my organization and want us to succeed." Participants’ responses were measured using a five-point Likert scale extending from (1) "strongly disagree," (2) "disagree," (3) " natural," (4) "agree, too, (5) " strongly agree," and there is no any converse scored items.

The overall score of the spiritual leadership questionnaire and its dimensions graded as follows: scoring equal to or more than 75% indicated high agreement on the overall spiritual leadership questionnaire and its dimensions, scoring ranging from 50% to less than 75% revealed moderate agreement, while scoring less than 50% was considered low agreements. The Cronbach's Alpha reliability of the original version of the scale is 0.98. The Arabic Version of SLQ confirmed its reliability and suitability for the advanced analysis as Cronbach's Alpha was 0.96 [1].

#### Section III: passion scale

This scale originated from Vallerand et al. in English. It is a self-reported scale composed of 14 items in which 7 measure harmonious passion (HP); this dimension emphasizes an active perspective where the person has control over the work, personal desire allows them to engage in the work thoroughly, and it is in harmony with the person’s other activities. The instance of item incorporated, "The new things that I discover with this activity allow me to appreciate it even more." The remaining 7 assess obsessive passion (OP), which highlights a passive perception where the person feels compelled to participate in the work, the work takes a proportion of space in the person’s self, and conflict with other aspects of one’s life is experienced. Examples of items involved are "I have difficulty imagining my life without this activity" and "My mood depends on me being able to do this activity."

Each item is ranked by participants along a seven-point continuum scale, extending from (1) "do not agree at all" to (7) "completely agree." There are no reverse-scored items. The score of the overall items was summed up, and the whole was distributed by the number of the items, giving a mean score of passion for work and its dimensions. A higher score corresponds to a higher tendency towards a passion for work among the studied nurse colleagues. A total score ranged from 14 to 98, and each dimension extended from 7 to 49. The scores were converted into percentage scores. The original version of the passion scale reflected validity and remarkable internal consistency with Cronbach's alpha; the internal consistencies of the two dimensions, including harmonious passion and obsessive passion, were 0.84 and 0.91, respectively [17].

#### Section V: the 9- Self-Report Altruism Scale (9- SRA)

The 9-Self-Report Altruism Scale (9-SRA) was developed by Manzur and Olavarrieta [25]. In English to assess altruism. It is a simplified version of the original SRA scale developed by Rushton et al. [26]. The items included “I have given money to a charity" and "I have offered my seat to a stranger standing. " It is a 9-item instrument with five response options comprising never, once, more than once, often, and very often. Each item is scored on a 5-point Likert scale from 0 to 4. The score of the whole items was summed up, and the entire was distributed by the number of the items reflecting a mean score of altruism; a higher score indicates the extent to which the studied nurse colleagues engaged in altruistic behaviors. A total score ranged from 0 to 36, which was transformed into a percentage score.

The 9-SRA confirmed satisfactory reliability and validity and represented a more parsimonious instrument to assess altruism. The scale reliability was assured as Cronbach's alpha coefficient was judicious, where α = 0. 77 [25].

### Questionnaires’ translation, its validity, and reliability

To guarantee the linguistic and conceptual accuracy of the questionnaires utilized, plus confirm cultural relevance to Arabic-speaking contributors, the questionnaires' translation and back-translation process was precisely accomplished.

Primarily, the questionnaires were translated from English to Arabic using the forward-and-backward translation technique. This comprised two independent translators translating the questionnaires from English to Arabic; this step guaranteed a preliminary translation identical to the original, considering linguistic differences. Followed by another two translators for the back translation from Arabic.

to English. Fluency in both languages, knowledge of the terminology utilized in the academic field, and familiarity with cross-cultural research methodologies were among the requests to choose the bilingual translators for the back-translation. These requirements were fulfilled by the selected translators, who contributed a profundity of knowledge to the translation process that made it easier to communicate complex concepts accurately.

The versions were compared after being back-translated, and differences ranging from minor linguistic variations to more significant conceptual misalignments were detected. These disparities were addressed through iterative revisions, guaranteeing a high level of equivalency with the original scales. This iterative translation process made it possible to carefully examine linguistic subtleties and cultural sensitivities, improving the questionnaire's lucidity and suitability for the intended participants.

Moreover, to evaluate the expert validity of the questionnaires, the translations were critically reviewed by a panel of conversant nursing professionals from the Psychiatric Nursing, Mental Health, and Nursing Administration departments, who offered input on its whole breadth, usability, and applicability. They were chosen for their dual expertise in nursing education and bilingualism. Criteria for their selection encompassed their academic credentials (Ph.D. in Nursing), English-language publications, and background in cross-cultural research. Specific changes were made due to their valuable contribution, including clarifying potentially confusing items and adapting some terms to reflect regional cultural contexts better. For the contemporary study, using SPSS version 26.0, Cronbach’s alpha was calculated to affirm the reliability of the utilized questionnaires, yielding results of 0.95 and 0.93 for the SLQ and the 9- SRA correspondingly. Likewise, Cronbach's alpha values of 0.96, with 0.86 for HP and 0.88 for OP, revealed a commendable internal consistency for the Passion Scale and its dimensions.

### Pilot study

Pilot research was carried out on 10% of all the nurse colleagues being surveyed (*n* = 39) who were chosen randomly. The pilot study acted as a last verification for comprehensibility and cultural suitability, appraised the time required to complete the instruments, and tested their clarity, applicability, and possibility. Furthermore, finding any hurdles or concerns can impede data collection. Nurse colleagues who shared in the pilot study were excluded from the entire research sample to guarantee the reliability of the results. Grounded on the results of the pilot study, no adjustments were made. The study tools were clear and vibrant.

### Data collection phase

Official permissions from the authorities of the aforementioned settings were obtained to confirm their collaboration and guarantee that the study would be conducted after duly illuminating its inducement. The researchers interviewed nurse colleagues who met the eligibility criteria and delivered their informed verbal consent after clarifying the purpose and nature of the study to gain their abundant collaboration. The self-report instruments were filled out by the nursing colleagues individually.

### Ethical considerations

The research proposal was permitted ethically by the Research Ethical Committee of the Faculty of Nursing, Sohag University (No. 155, 5–12–2023). The studied nurse colleagues provided informed consent after clearly illuminating the study's aim. The researchers confirmed that all data were utilized exclusively for research purposes. Anonymity and confidentiality were respected and reflected by assigning a code number to each questionnaire. Besides, the right to refuse to participate or withdraw from the study even after beginning at whatever time without fronting any undesirable ramifications was guaranteed. Finally, the workflow in the settings above was not perturbed due to the data collection process.

### Statistical analysis

Data analysis was performed using SPSS 26.0 (IBM Inc., Chicago, IL, USA) to examine the survey responses from the 391 recruited nurse colleagues. Numbers and percentages were used to describe the qualitative data. After performing the Kolmogorov–Smirnov test to ensure the data were normally distributed, quantitative data were pronounced using means and standard deviations. The attained results were considered significant if the *P*-value was equal to or less than 0.05 and 0.01. Pearson's correlation analysis evaluated the correlations between spiritual leadership, passion, and altruism among nurse colleagues. Multiple linear regression analysis with R^2^ calculation was done to assess factors predicting spiritual leadership among nurse colleagues.

## Results

Table 1 Presents nurses' baseline characteristics; 391 nurses participated in this study. The majority (88%) of the participants were female, (70.8%) were married, and less than half (43.0%) of them had a Bachelor's degree in nursing. More than half (55.5% 57.5%) were practical guides and had less than five years of experience in the same specialty, respectively. Also, (52.2%) of them lived in urban areas and (30.7%) in upper Egypt.

**Table 1:** Personal characteristics of the studied nurse colleagues (*n* = 391)

Personal Characteristics	Categories	No	%
**Gender**	Male	47	12.0
	Female	344	88.0
**Marital status**	Single	88	22.5
	Married	277	70.8
	Divorced	7	1.8
	Widow	17	4.3
**Educational Level**	Bachelor's degree in nursing	168	43.0
	Diploma	31	7.9
	Master degree	103	26.3
	Doctorate	89	22.8
**Specialty**	Practical guide	217	55.5
	Clinical demonstrator	60	15.3
	Assistant lecturer	45	11.5
	Lecturer	36	9.2
	Assistant professor	26	6.6
	Professor	7	1.8
**Years of experience**	< 5	225	57.5
	5 < 10	122	31.2
	10 < 15	20	5.1
	≥ 15	24	6.1
**Residence**	Urban	204	52.2
	Rural	187	47.8
**Geographic area**	Upper Egypt	120	30.7
	Eastern Egypt	54	13.8
	Western Egypt	89	22.8
	Delta Egypt	70	14.8
	Center Egypt	58	17.9

Table 2 Reveals a detailed insight into participants' mean scores and distribution across different levels for the studied variables of spiritual leadership, passion, and altruism. In terms of spiritual leadership, the participants exhibited a mean score of 60.77 ± 13.659, with (50.9%) of them falling into the "high" category, specifically (61.4%) scoring in the "high" category for hope/faith. Moving on to the passion-related variables, the total passion mean score was 68.16 ± 20.527, and the harmonious passion mean score was 34.98 ± 10.46, with 49.9% of them in the "high" category. Also, obsessive passion had a mean score of 33.17 ± 11.12, with (48.8%) percentage in the "high" category. Finally, the total altruism mean score of 29.45 ± 9.095, with (40.2%) of them having high altruism.

**Table 2:** Mean scores and levels of the studied variables (Spiritual leadership, passion, and altruism) among the studied nurse colleagues (*n* = 391)

**Study Variables**	**M** ± **SD**	**High** **No (%)**	**Moderate** **No (%)**	**Low** **No (%)**
**Total Spiritual Leadership**	60.77 ± 13.659	199 (50.9)	90 (23.0)	102 (26.1)
✓ Vision	18.28 ± 4.250	214 (54.7)	86 (22.0)	90 (23.0)
✓ Hope/faith	19.12 ± 4.547	240 (61.4)	75 (19.2)	65 (16.6)
✓ Altruistic love	23.37 ± 6.196	147 (37.6)	126 (32.2)	118 (30.2)
**Total passion**	68.16 ± 20.527	186 (47.6)	93 (23.8)	112 (28.6)
✓ Harmonious passion	34.98 ± 10.46	195 (49.9)	100 (25.6)	96 (24.6)
✓ Obsessive passion	33.17 ± 11.12	190 (48.6)	64 (16.4)	137 (35.0)
**Total altruism**	29.45 ± 9.095	157 (40.2)	101(25.8)	133 (34.0)

Table 3, Demonstrates that (89.5%) of participants have a strong spiritual relationship with God, (84.1%) of them engaged in religious or spiritual practices such as prayer, fasting, or almsgiving, (57.8%) of them hadn't a spiritual teacher or spiritual director guiding them, (64.7%) of them felt that they had a strong connection with nature or are connected to its spirituality, (55.2%) of them did not connect with the self through practices such as meditation or yoga. Furthermore, all participant's spiritual data had significant differences with spiritual leadership**,** passion, and altruism at (*P* < 0.05).

**Table 3:** Relation between study variables: Spiritual leadership, passion, and altruism and nurse colleagues’ spiritual data (*n* = 391)

Spiritual Data	No (%)	Spiritual Leadership		Passion		Altruism	
		**M** ± **SD**	**t/F (P)**	**M** ± **SD**	**t/F (P)**	**M** ± **SD**	**t/F (P)**
**Have a strong spiritual relationship with God**							
Yes	350 (89.5)	61.98 ± 13.02	5.309 .000	69.32 ± 20.0	3.297 .001	30.38 ± 8.68	6.182 .000
No	41 (10.5)	50.41 ± 14.69		58.29 ± 22.49		21.51 ± 8.70	
**Engage in religious or spiritual practices such as prayer, fasting, or almsgiving**							
Yes	329 (84.1)	63.58 ± 12.40	10.654 .000	71.78 ± 18.35	8.768 .000	31.19 ± 8.31	9.707 .000
No	62 (15.9)	45.83 ± 9.76		48.98 ± 20.91		20.20 ± 7.35	
**Have a spiritual teacher or spiritual director guide you**							
Yes	165 (42.2)	63.10 ± 12.40	2.907 .004	71.96 ± 19.47	3.164 .002	31.96 ± 8.26	4.799 .000
No	226 (57.8)	59.07 ± 14.29		65.39 ± 20.87		27.61 ± 9.25	
**Feel that you have a strong connection with nature or are connected to its spirituality**							
Yes	253 (64.7)	63.72 ± 12.80	6.036 .000	72.34 ± 19.33	5.664 .000	32.35 ± 7.750	9.476 .000
No	138 (35.3)	55.36 ± 13.56		60.50 ± 20.50		24.12 ± 8.99	
**Connect with the self through practices such as meditation or yoga**							
Yes	175 (44.8)	63.31 ± 12.84	3.352 .001	71.83 ± 19.85	3.216 .001	31.92 ± 8.25	4.978 .000
No	216 (55.2)	58.71 ± 13.97		65.19 ± 20.63		27.44 ± 9.27	

Table 4 Results disclose significant and positive correlations between spiritual leadership, passion, and altruism as follows (*r* = 0.644, *P* < 0.001), (*r* = 0.519, *P* < 0.001), and (*r* = 0.509, *P* < 0.001) correspondingly.

**Table 4:** Correlation between spiritual leadership, passion, and altruism among the studied nurse colleagues (*n* = 391)

Pearson's Correlations	Pearson's r	p
Spiritual leadership ➜ Passion	0.644***	< .001
Spiritual leadership ➜ Altruism	0.519***	< .001
Passion ➜ Altruism	0.509***	< .001

Note (r): Pearson coefficient * *p* < .05, ** *p* < .01, *** *p* < .001

**Fig. 2:**
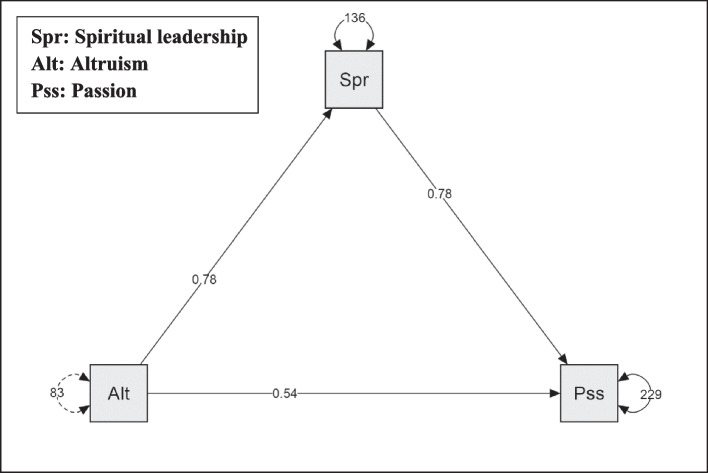
Mediation analysis model with altruism as the independent variable, spiritual leadership as the mediator, and passion as the dependent variable

Figure 2 and Table 5 illustrate the mediation analysis results of spiritual leadership regarding the relationship between altruism and passion. These findings confirm the positive direct effects between altruism and passion with (B = 0.540, *P* < 0.0001) and altruism to spiritual leadership (β = 0.780, *P* < 0.0001), also between spiritual leadership to passion (B = 0.781, *P* < 0.0001). In addition, these findings clarify the indirect effect path between altruism, spiritual leadership, and passion (B = 0.608, *P* < 0.0001).

**Table 5:** Path analysis, direct and indirect effects of the study variables

Model Pathways	Estimate	S.E	t/z-value	P	95% Confidence Interval	
	**(B)**				**Lower**	**Upper**
Altruism ➜ Passion	0.540	0.098	5.482	< .001	0.347	0.733
Altruism ➜ Spiritual leadership ➜ Passion	0.608	0.072	8.454	< .001	0.467	0.750
Spiritual leadership ➜ Passion	0.781	0.066	11.858	< .001	0.651	0.910
Altruism ➜ Spiritual leadership	0.780	0.065	11.978	< .001	0.652	0.907

B: Unstandardized regression coefficients

*S.E* Standard errors

## Discussion

Spiritual leadership is a concept that is often overlooked in the healthcare profession but can profoundly impact how nurses approach their work. Considering the associative analysis of spiritual leadership with passion and altruism in patient care is crucial for nurse colleagues. By tapping into spiritual beliefs and values, nurses can find a more profound sense of purpose and meaning in their work, leading to increased compassion and empathy towards patients and a stronger connection with colleagues and the healthcare team. Incorporating spiritual leadership into nursing practice enhances the ability to provide holistic care and support to those in need. Striving to embody spiritual leadership qualities can create a nurturing and healing environment for both patients and nurses [1]. Therefore, this study aimed to investigate the role of spiritual leadership in the relationship between altruism and passion for work among nurse colleagues.

One of the imperative results of the current study was that the nurse colleagues generally perceive spiritual leadership positively and highly, with a majority falling into the high category for hope or faith, which reflects a strong inclination towards these aspects of spiritual leadership. This could be attributed to the high levels of perceived spiritual leadership among nursing colleagues that align with the core values of nursing, which emphasize holistic, compassionate care. This spiritual approach can enhance resilience in nurses by offering meaning and inner strength to navigate the profession's challenges.

Analogous with the foregoing findings, a recent study done by Abouzaid [27], reported that the total levels of spiritual leadership among nurse leaders were high. As well, Ali et al. [28], revealed that nursing managers’ spiritual leadership levels were high on the level of meaning/calling, vision, and hope/faith dimensions, while the largest percentage of participants who scored an individual subscale as “low level” was for the “altruistic love” subscale. Conversely, the previous findings were in disagreement with a study conducted by Abou Zeid et al. [1], which revealed that nursing educators perceived their spiritual leadership behaviors at a moderate level.

One important factor that is connected to the quality of work-life is a passion for one's job. It promotes flow at work, which seems to be good for nurse colleagues' mental health and guards against burnout [29]. In terms of the overall mean score and the levels of passion among nurse colleagues, more than half exhibited a high level of passion. The highest mean score was associated with harmonious passion, linked to positive outcomes such as improved well-being and effective coping strategies. The intense nurse colleagues’ passion fosters dedication, energy, and a sense of purpose. The findings indicate that colleagues have a healthy, harmonious passion for their work.

This is not surprising, considering the difficult working conditions that nurse colleagues face, such as an underappreciated and unsupportive work environment, having little time for professional development, and juggling a demanding workload. This can be explained by the fact that nurse colleagues have a sense of belonging and a strong commitment to do their work, without hesitation, even if they are overworked or in danger [30]**.** Their acceptance of their mission and ethical commitment allowed them to play a crucial role in promoting their work with passion. In the same track, a study carried out by Li et al. [31], clarified a positive association between leaders’ and workers’ passion at work; a leader’s work passion was transferred to employees via emotional contagion. Leaders may act as role models in fostering passion at work. On the contrary, Luo et al. [32], revealed that nurse colleagues expressed that they didn’t have passion for work.

As for the total mean score of altruism; the study findings indicated a highly positive perception of altruistic behaviors. Interestingly, participants appear to be more evenly distributed across the "High," "Low," and "Moderate" categories for altruism. This suggested a varied engagement in altruistic acts among the participants. The possible explanation for these findings might be due to that the nurse colleagues are familiar that altruism is a cornerstone of the nursing profession. They select to track a nursing profession since they are altruistically oriented, wish to work and comfort others, or consider nursing a significant vocation; often, it is the first and merely educational choice. Conversely, the previous findings were in disagreement with those of a study conducted by Chen et al. [19], who indicated that altruism has been decreasing over time. In this regard, Bhuvana & and Pavithra [22] revealed that altruism has been decreasing among health professionals.

One of the conspicuous results of the current study is that notably, spiritual leadership had a statistically significant positive correlation with passion and altruism among nurse colleagues. This suggests that higher levels of spiritual leadership are associated with increased passion, as well as heightened altruistic tendencies among nursing colleagues. This may be explained by the fact that one promising strategy for fostering a positive work environment is spiritual leadership; spiritual leaders help their followers develop a strong calling by fostering a clear and inspiring vision, bolstering their beliefs, and showing compassion for others, work-based spiritual leadership initiatives yield excellent results. In this regard, a healthier workplace and an atmosphere where people can express themselves freely and be passionate are the results of spiritual leaders' empathy and regard for their staff, their attention to both positive and negative feedback during interactions with them, and their best efforts to meet their needs [33, 34].

The results of the current study revealed that altruism is one of the most significant attributes of spiritual leadership in nursing. Few studies have been carried out concerning the interrelation between passion, leadership, and spirituality. Anane [35] is one of the few researchers who has studied the relationship between spiritual leadership and extrinsic passion and found that spiritual leadership (altruism and membership) is effective with the combination of extrinsic passion on organizational commitment.

The eventual goal of the existing work was to investigate the role of spiritual leadership in the relationship between altruism and passion for work among nurse colleagues. Moving to the mediation analysis results, the results verified that spiritual leadership has both direct and indirect effects on nurses' passion and altruism. Spiritual leadership directly impacts passion, and passion, in turn, mediates the relationship between spiritual leadership and altruism.

These indirect effects provide important insights into the interplay between these variables. The findings suggest that spiritual leadership has a profound influence on cultivating altruism and passion among nurse colleagues. Spiritual leadership inspires nurse colleagues to go beyond their professional duties and extend genuine compassion and empathy. It fosters a supportive, nurturing work environment where nurse colleagues are motivated to collaborate and uplift one another. Spiritual leadership ignites a collective passion for providing holistic, compassionate care, creating a sense of unity and shared purpose. It also instills resilience and inner strength, enabling followers to navigate challenges with grace. Overall, the influence of spiritual leadership helps establish a culture of altruism and passion, ultimately enhancing the quality of patient care. Research studies suggested that spiritual leadership values can stimulate and inspire employees to increase loyalty, altruistic behaviors, passion, and active participation, ultimately enhancing employee commitment to the organization [11, 36, 37].

Our study represents a novel attempt to examine spiritual leadership as a mediating factor between altruism and passion. Without the least suspicion, this existing study is notable for equally theoretical and clinical implications because it sheds light on the significance and necessity of taking spiritual leadership into account as a factor contributing to nurse colleagues' tendency towards passion for work and altruistic behaviors. Emphasizing the mediating role provides novel implications in nursing [38–40]. Understanding the incentives of nurse colleagues' propensity towards altruism and passion is essential to intensify them. Subsequently, it has significant favorable outcomes on their physical, psychosocial, and emotional well-being, and in turn is linked to the quality of work life, since one of the professional principles that best represents the quality of work is altruism, which is characterized by a concern for the well-being of others and a readiness to prioritize and make sacrifices for their needs. The results of this study may guide decision-makers to create plans for enhancing spiritual leadership among nursing colleagues through training, education, and policy development.

### Implications for nursing

Conferring to the findings of the current study, spiritual leadership plays a critical role in fostering nurse colleagues’ altruism and passion for work. Consequently, our research confirms that cultivating workplace spirituality on a daily agenda is a key component of management that requires spiritual competencies from leaders, which ultimately enhance passion for work, performance, and altruistic behaviors. In addition, fostering a culture of hope, faith, and altruism can improve team morale and collaboration, and benefit organizational outcomes. Carrying out periodical training programs for leaders to promote spiritual leadership behaviors and consideration should be fulfilled. Moreover, the study results highlight the need for developing approaches to raise commitment, intensify nurse colleagues’ satisfaction, and generate a positive work environment to improve passion for work among nurse colleagues. Educators and decision-makers must emphasize the development of harmonious passion and altruistic behaviors to cultivate well-rounded and empathetic nursing professionals. They should integrate spiritual leadership theories into undergraduate and postgraduate nursing curricula. Upcoming studies are essential to be fulfilled by employing large random samples to advance awareness about factors that inspire altruism and passion. Also, it may be valuable for future research to study diverse types of leadership that may be mainly malevolent about stream in the work situations.

## Conclusion

In deduction, the current study results verified that nurse colleagues had high levels of perceived spiritual leadership, passion, and altruism. The results revealed noteworthy positive correlations between spiritual leadership, passion, and altruism. Furthermore, mediation analysis highlighted that spiritual leadership could be mediating the effect of altruism on the passion level. This study delivers valued insights into the crucial role of spiritual leadership in augmenting nurse colleagues’ altruistic tendencies and passion. Our research confirms the prominence of cultivating and implementing spiritual leadership in nursing educational institutions. Besides, educational institutions should take into consideration the most effective leaders who promote workplace spirituality, which eventually, in return, enhances the altruism, passion, and work engagement of nurse colleagues.

## Supplementary Information

Supplementary Material 1.

## Data Availability

No datasets were generated or analysed during the current study.

## Abbreviations

SLQ: Spiritual Leadership Questionnaire
SRA: Self-Report Altruism
