# Supplementary material for: Exploring the role of spiritual leadership among nurse colleagues: an associative analysis of its impact on passion and altruism
Source: BMC Nurs. 2025 Feb 7;24:142. doi: 10.1186/s12912-025-02750-5 (PMC11804073; doi:10.1186/s12912-025-02750-5)
Supplement: Supplementary file 1 — Supplementary Material 1. [file 12912_2025_2750_MOESM1_ESM.docx]

**Nurse’ Personal and Job-related Data Questionnaire:**

| **Questions** | **Choices** |
| --- | --- |
| Gender | Male |
|  | Female |
| Marital status | Single |
|  | Married |
|  | Divorced |
|  | Widow |
| Educational Level | Bachelor's degree in nursing |
|  | Diploma |
|  | Master degree |
|  | Doctorate in Nursing |
| Specialty | Nurses |
|  | Clinical demonstrator |
|  | Assistant lecturer |
|  | Lecturer |
|  | Assistant professor |
|  | Professor |
| Years of experience | < 5 |
|  | 5 < 10 |
|  | 10 < 15 |
|  | ≥15 |
| Residence | Urban |
|  | Rural |
| Geographic area | Upper Egypt area |
|  | Eastern Egypt area |
|  | Western Egypt area |
|  | Delta Egypt area |
